# ﻿*Clermontiahanaulaensis* (Campanulaceae, Lobelioideae), a new, critically endangered species from Maui, Hawaiian Islands

**DOI:** 10.3897/phytokeys.227.100725

**Published:** 2023-06-19

**Authors:** Hank Oppenheimer, David H. Lorence, Warren L. Wagner

**Affiliations:** 1 Plant Extinction Prevention Program, Pacific Cooperative Studies Unit, University of Hawaii, PO Box 909, Makawao, HI 96768, USA University of Hawaii Makawao United States of America; 2 National Tropical Botanical Garden, 3530 Papalina Road, Kalaheo, HI 96741, USA National Tropical Botanical Garden Kalaheo United States of America; 3 Department of Botany, Smithsonian Institution, P.O. Box 37012, Washington, DC, 20013-7012, USA Smithsonian Institution Washington United States of America

**Keywords:** Campanulaceae, *Clermontia*, conservation, endemism, Hawaiian Islands

## Abstract

*Clermontiahanaulaensis* H.Oppenheimer, Lorence & W.L.Wagner, **sp. nov.**, a newly discovered, narrowly distributed endemic species, is herein described based on its morphological characteristics and illustrated with field photos and a line drawing. It is currently known only from the slopes of Hana‘ula, in Pōhākea Gulch, Mauna Kahālāwai, west Maui, Hawaiian Islands. It differs from all other species of *Clermontia* Gaudich. by the combination of its (2)3–4(–5) flowered inflorescence, violet colored perianth often suffused with creamy white streaks or sometimes creamy white with violet-purple irregular veins, (30)35–45(–50) mm long, perianth tube 15–25(–27) mm long, 9–10 mm wide, the lobes 20–26 mm long, (2–)3–3.5 mm wide, with petaloid calyx lobes 1/2–4/5 as long as the petals. A key to the *Clermontia* species and subspecies occurring on Maui is provided. Its habitat is described. Its conservation status is proposed as critically endangered (CR), and conservation efforts are discussed.

## ﻿Introduction

The Hawaiian lobeliads (Campanulaceae, Lobelioideae) are the largest plant clade restricted to any archipelago, and originated from a single introduction ca. 13 mya (Givnish et al. 2009). In the most recent monograph of the genus *Clermontia* Gaudich., 22 species and nine non-autonymic subspecies were recognized (Lammers 1999). *Clermontia* occurs as terrestrial or epiphytic shrubs or small trees on the six largest Hawaiian Islands from 150 m to 2100 m elevation in mesophytic to wet forests, cloud forests, bogs, and shrublands. Lammers (1995) argued, based on a morphological analysis of relationships within *Clermontia*, that the genus arose on Maui rather than Kaua‘i or older islands. However, Givnish et al. (2013) used plastid and nuclear DNA sequence data to show that *Clermontia* did arise on Kaua'i or an older island and largely obeyed the progression rule, with inferred inter-island dispersal events largely down the Hawaiian chain, from older to younger islands. Kaua‘i, the oldest current high island is estimated to be 5.1 mya, and has just a single species of *Clermontia*. Hawai‘i Island on the other hand is <1 mya and is home to 11 species (Lammers 1995, 1999). Maui has 14 taxa (not including *C.hanaulaensis*). One is presumed extinct (*C.multiflora* Hillebr.), while C.peleanaRocksubsp.singuliflora (Rock) Lammers is apparently extirpated on east Maui, but is still extant on Hawai‘i Island. *Clermontialindseyana* Rock is included in this tally based on two specimens at the Bernice P. Bishop Museum Herbarium [BISH], although populations on the leeward slope of Haleakalā are probably not this taxon, but may instead be *C.kakeana* Meyen (R. Pender, pers. comm.). Therefore, additional studies are needed to determine if *C.lindseyana* occurs on Maui.

**Figure 1.**
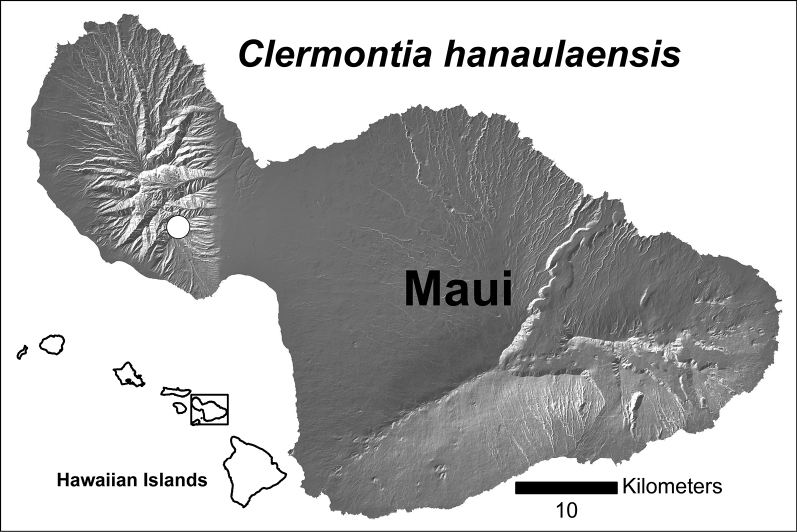
Distribution map of *Clermontiahanaulaensis* on Maui (white dot).

During the course of rare plant field work in the mountains of west Maui, plants belonging to the genus *Clermontia* were found across several small ridges and gullies on the slopes below Hana‘ula, in the Pōhākea Gulch drainage basin (Fig. 1). Initially only eight mature individuals and several immature plants were observed. These plants were not assignable to any taxa known from Mauna Kahālāwai, or elsewhere on Maui. Three possibilities were considered.

The first possibility considered was that these plants represented natural hybrids. Three congeners occur sympatrically in the study area: the at-risk, but locally common C.arborescens(H. Mann)Hillebr.subsp.arborescens, endemic to Mauna Kahālāwai; C.grandifloraGaudich.subsp.grandiflora, also endemic to Mauna Kahālāwai, but not considered to be rare; C.grandiflorasubsp.munroi (H. St. John) Lammers, which is less common locally, but one of the most widespread of all taxa in the genus, occurring on east and west Maui, Lana‘i, and Moloka‘i. However, the newly discovered plants do not resemble any of these three taxa as might be expected if they are hybrids, as they differ in the height of the plants, size and texture of the flowers, the length of the peduncles, the number of flowers per inflorescence, and the relative length of the calyx lobes compared to the corolla, among other features. The discovery of many more plants during subsequent surveys tends to negate the possibility that these plants could be hybrids as there are nearly 100 mature individuals and several dozen immature plants and seedlings occurring over an area of 0.1 km^2^.

The second possibility considered these plants to potentially represent a new distributional record for a species previously known from another island. However, there have been no new records documented for the genus *Clermontia*, and only a single new distributional record exists for Hawaiian Campanulaceae, namely *Lobeliahillebrandii* Rock (Lorence et al. 1995; Wagner et al. 1999, 2005). This possibility was discarded after unsuccessful attempts to key it out using the keys in Wagner et al. (1999) and Lammers (1991). Furthermore, images were sent to botanists at the Hawai‘i Branch of the Division of Forestry and Wildlife (DOFAW) who did not recognize the new plants (Josh VanDeMark and Lyman Perry pers. comm.). Finally, flowers of *C.hanaulaensis* were compared to verified images of other *Clermontia* taxa on Richard Pender’s Flickr website (https://flickr.com/photos/123604592@N07/) which also did not result in a match.

The final possibility remaining was that these plants represent a new, undescribed taxon. Although new species of *Cyanea* Gaudich. continue to be discovered and described (Oppenheimer and Lorence 2012; Spork-Koehler et al. 2015; Oppenheimer 2020), only one new subspecies of *Clermontia* has been described in the past 30 years (e.g., C.grandiflorasubsp.maxima Lammers). No new taxon at full specific rank has been described and accepted since *C.lindseyana* Rock was published in 1962 (Rock 1962). Study of the material collected, herbarium vouchers, literature, field observations, and photographs revealed these plants are morphologically distinct from all other known taxa and represent a new species, herein described.

## ﻿Methods

All measurements given herein are taken from dried herbarium specimens unless otherwise noted. Certain features, such as shapes and colors, were supplemented with information from field notes and photos. Measurements are presented in the description as follows: dimensions followed by units of measurement (mm, cm, m). All specimens cited have been seen by the authors and are deposited at the herbaria cited herein. The area of occupancy (AOO) was calculated based on field observations and herbarium collection data, and the conservation status is proposed following the IUCN Red List Category criteria Version 14 (IUCN Standards and Petitions Committee 2019; http://www.iucnredlist.org/documents/RedListGuidelines.pdf).

## ﻿Taxonomic treatment

### ﻿ Clermontia hanaulaensis

Taxon classificationPlantaeColeopteraCerambycidae

H.Oppenheimer, Lorence, & W.L.Wagner sp. nov.

9BDF90BE-4F19-54E1-9A8E-850165BC48CA

urn:lsid:ipni.org:names:77321536-1

#### Type.

**Figure 2.**
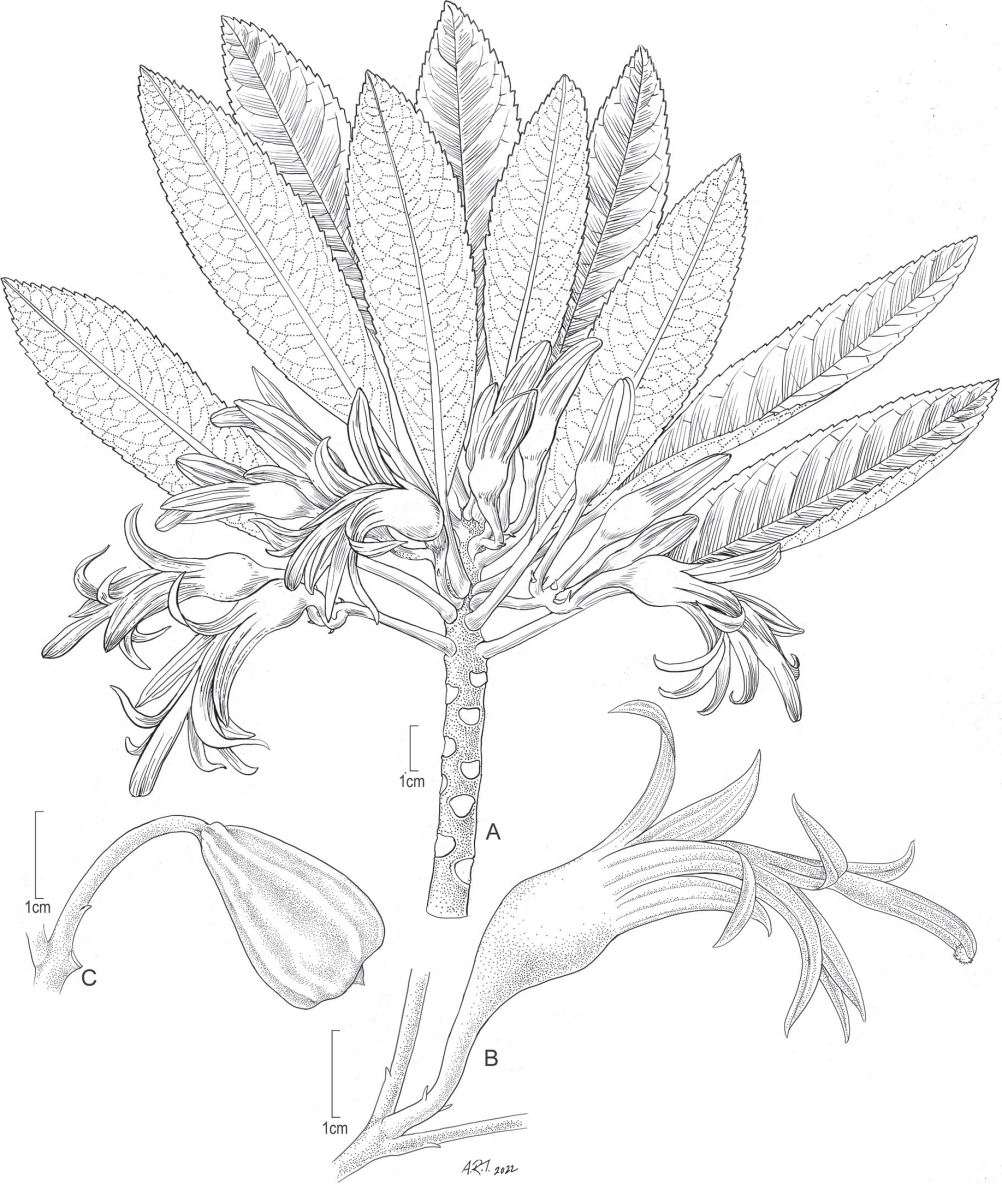
*Clermontiahanaulaensis* H.Oppenheimer, Lorence & W.L.Wagner **A** habit of flowering stem **B** detail of flower at anthesis **C** fruit. (**A, B**, drawn from type collection and field images of type plant (*Oppenheimer & Severson H82102*; isotype US) **C** drawn from field image of non-type plant in Pōhākea Gulch). Illustration by Alice Tangerini.

**Figure 3.**
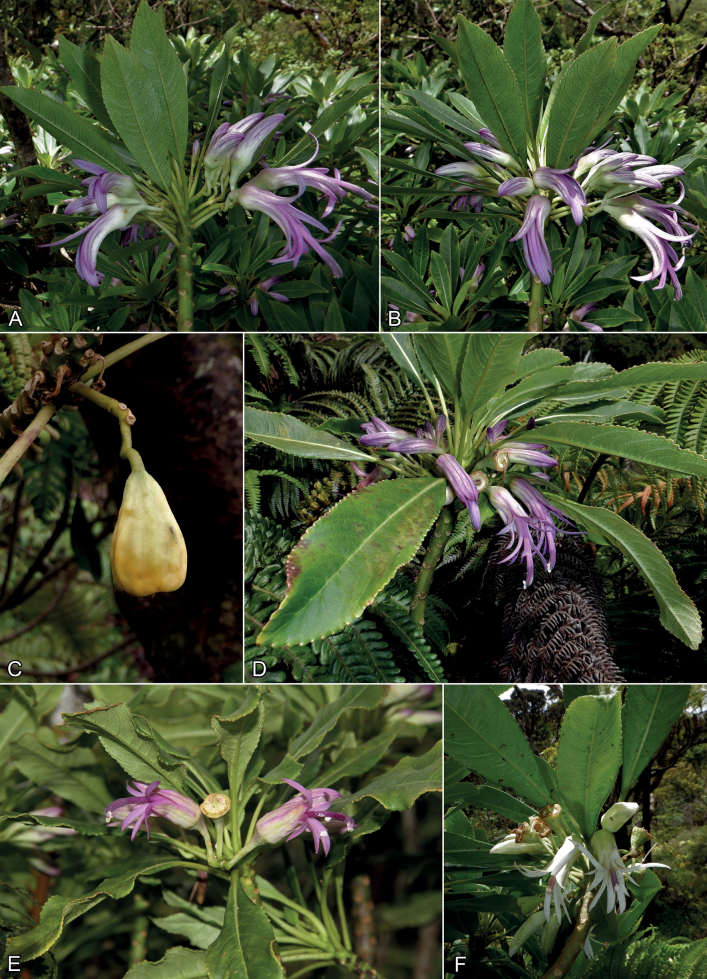
*Clermontia* from Pōhākea Gulch area, west Maui **A–C***Clermontiahanaulaensis* H. Oppenheimer, Lorence & W.L. Wagner **A, B** habit, from field images of type collection with purple and white perianth (from *Oppenheimer & Severson H82102*) **C** mature fruit, taken in Sep. 2020 **D–F** from other *Clermontia* plants in Pōhākea Gulch showing variations that could represent hybridization between *C.hanaulaensis* and other sympatric species or between other species in the area **D** habit, form with shorter, wider calyx showing slight separation of calyx tube from corolla tube, taken in Sep. 2020 (unvouchered) **E** habit, form with shorter, wider calyx, taken in Aug. 2020 (unvouchered) **F** habit, form with white perianth, taken in Aug. 2020 (*H. Oppenheimer & K. Severson H82101*, BISH, PTBG, US). All photos by H. Oppenheimer.

**USA. Hawaiian Islands: Maui**: west Maui, Wailuku District, slopes of Hana‘ula, Pōhākea Gulch, ca. 1183 m, 4 Aug 2021, *H. Oppenheimer & K. Severson H82102* (Holotype: PTBG [PTBG1000093350]; Isotypes BISH, US). Figs 2, 3.

#### Description.

Shrubs or small trees up to 3 m tall, flowering at 1.5–3 m tall, terrestrial, branched from near base, with repeated candelabra-like branching, bark rugose-fissured, light brown, leafy branches green, latex white. Leaves clustered at the distal ends of the branches, alternate, with short internodes, simple, petiolate; lamina 10–12(–18) cm long, 2.0–3.5(–4) cm wide, narrowly elliptic to oblanceolate, chartaceous; adaxial surface green, glossy when fresh, drying dull, glabrous; abaxial surface paler than adaxial surface, glabrous, secondary veins 15–16 on each side; margins entire in basal ¼, otherwise callose-crenulate; apex acute to short-acuminate, occasionally with a short mucro 1 mm long; base cuneate to attenuate; petiole 3–4(–6) cm long, glabrous; seedling leaves pubescent. Inflorescence (2)3–4(–5)-flowered, glabrous; flowers 5-merous; peduncle 15–30(–42) mm long; bracts triangular, ca. 1 mm long, deciduous; pedicels 8–18 mm long; bracteoles basal, 1.0–1.2 mm long, narrowly lanceolate, acute to acuminate, sometimes short mucronate, ciliate; hypanthium obconic or hemispheric, green, ca. 7–10 mm long, 8–10 mm wide; corolla weakly zygomorphic to nearly rotate when fresh, slightly to moderately curved, perianth (30–)35–45(–50) mm long, perianth tube 15–25(–27) mm long, 9–10 mm wide, the lobes 20–26 mm long, (2.0–)3.0–3.5 mm wide, the dorsal and ventral lobes spreading in distal half, pale violet-purple, often suffused with creamy white streaks, occasionally creamy white, glabrous; calyx 1/2–4/5 as long as petals, lobes petaloid, similar in color to corolla, often pale greenish tinged toward base, sometimes creamy white with violet-purple irregular veins, lobes connate for 1/3–1/2 their length, not appressed to petals, erect to spreading; staminal column violet, 2.0–2.5 mm wide, filaments 30–40 mm long, anthers darker violet, anther tube 9–12 mm long, 2.3–3.0 mm wide. Fruit dull orange, obconic to turbinate, 15–20 mm long, 10–15 mm wide, smooth, sepals and petals caducous. Seeds obovoid, slightly compressed, 0.5–0.6 mm long, 0.5 mm wide, testa dark brown, glossy, smooth.

#### Distribution.

*Clermontiahanaulaensis* is known only from a single population on west Maui in several small ridges and gullies on the slopes below Hana‘ula, in the Pōhākea Gulch.

#### Habitat and ecology.

*Clermontiahanaulaensis* occurs in *Metrosideros* Banks ex Gaertn. Montane Wet Forest (Wagner et al. 1999) at ca. 1158–1213 m elevation with an annual rainfall of ca. 2600–2900 mm. The common associated woody elements are species of *Cheirodendron* Nutt. ex Seem., *Clermontia* Gaud., *Coprosma* J.R. Forst. & G. Forst., *Cyrtandra* J.R. Forst. & G. Forst., *Hydrangea* L., *Ilex* L., *Kadua* Cham. & Schltdl., *Myrsine* L., *Perrottetia* Kunth, *Pipturus* Wedd., and *Psychotria* L. Pteridophyte genera include *Athyrium* Roth, *Cibotium* Kaulf., *Dicranopteris* Bernh., *Diplazium* Sw., *Dryopteris* Adans., and *Sadleria* Kaulf. that are prevalent and form a dense ground cover. *Freycinetiaarborea* Gaudich. is a widespread liana. Common epiphytes include species of *Adenophorus* Gaudich., *Asplenium* L., *Elaphoglossum* Schott ex J. Sm., and several herbaceous species of *Peperomia* Ruiz & Pav. The terrestrial sedge *Carexalligata* Boott is occasional. The herbaceous *Ranunculusmauiensis* A. Gray is a distinctive, but extremely rare element of this plant community.

Soil is of typical basaltic origin derived from the original shield-building Wailuku Volcanic Series (Stearns and MacDonald 1942). The average annual rainfall is approximately 2700 mm. (Giambelluca et al. 1986).

Clermontiaarborescenssubsp.arborescens occurs sympatrically throughout the range of *C.hanaulaensis*, whereas C.grandiflorasubsp.munroi is scattered although locally common, and C.grandiflorasubsp.grandiflora occurs at the upper perimeter of the population. Even further away in much wetter habitat with annual rainfall above ca. 2900 mm are populations of *C.micrantha* (Hillebrand) Rock, while *C.kakeana* occurs in lower, drier areas with annual rainfall below ca. 2600 mm. These taxa are readily distinguished morphologically from *C.hanaulaensis* by the characters given in the key to the Maui species below.

#### Phenology.

*Clermontiahanaulaensis* has been observed to be flowering from July through September, with fruit maturing from August through October.

#### Etymology.

The specific name honors Hana‘ula, a peak on southern Mauna Kahālāwai (aka west Maui Mountains). *Lit.* red bay (Pukui et al. 1966); + Latin suffix -*ensis*, indicating a place of origin or growth. The Hawaiian vernacular names ‘*ōhā wai*, ‘*ōhā*, *hāhā*, ‘*ōhāhā*, ‘*ōhā wai nui*, and ‘*ōhāhā wai nui* apply to other species of *Clermontia* (Pukui and Elbert 1986; Lammers 1991; Wagner et al. 1999).

#### Specimens examined

**(*paratypes*). USA, Hawaiian Islands. Maui**, Wailuku District, slopes of Hana‘ula, Pōhākea Gulch, 30 Jul. 2020, *H. Oppenheimer H72005* (BISH, PTBG, US); *H. Oppenheimer H72006* (BISH, PTBG); *H. Oppenheimer H72007* (BISH, PTBG, US); *H. Oppenheimer H72008* (BISH); *H. Oppenheimer H72009* (BISH); 21 Aug. 2020, *H. Oppenheimer & K. Bustamente H82005* (BISH, PTBG); *H. Oppenheimer & K. Bustamente H82006* (BISH); 28 Sep. 2020, *H. Oppenheimer & K. Severson H92014* (BISH, PTBG, flowers and mature fruit in alcohol); *H. Oppenheimer & K. Severson H92015* (PTBG); 2 Oct. 2020, *H. Oppenheimer H102002* (BISH), *H. Oppenheimer H102003* (BISH); 10 Sep. 2021, *H. Oppenheimer & Z. Pezzillo H92101* (BISH, PTBG, US).

## ﻿Discussion

### ﻿Affinities

Hillebrand (1888) recognized 11 *Clermontia* species with six varieties, divided into two sections: Section Genuinae with calyx lobes as long as the corolla or a little shorter, and Section Clermontioideae, with the calyx lobes shorter and persistent. Rock (1919), in his monograph of the Hawaiian species of Tribe Lobelioideae, maintained both of Hillebrand’s Sections and recognized 23 species with four infraspecies. These two divisions are consistent with later treatments, but Section Genuinae was nomenclaturally corrected to Section Clermontia by Lammers (1991). In the most recent monograph of the genus, Lammers (1991) divided the genus into two sections, each with three series for a total of six taxonomic subdivisions.

A re-analysis of Lammer’s morphological data by Givnish et al. (2013) shows that the two sections are not supported even by morphology when properly analyzed, with almost all resolution disappearing in the unweighted strict consensus tree. Furthermore, their analysis of plastid and nuclear sequence data demonstrates that petaloid sepals – almost certainly the product of a single homeotic mutation (Hofer et al. 2012; Givnish et al. 2013) – do not define a clade in *Clermontia*, with maximum-parsimony and Bayesian inference both showing multiple origins and losses of the trait. Similarly, Pender (2013) concluded that these entities were not fully supported by cpDNA. Givnish et al. (2013) demonstrated that the molecular data place *Clermontiafauriei* H. Lév. from Kaua‘i, and then *C.persicifolia* from West O‘ahu sister to all other *Clermontia* species (mostly from Maui Nui and Hawaii), pointing to an initial adherence to the progression rule. Finally, they show that *C.pyrularia* Hillebr. is actually a species of *Cyanea*. Hunter (2018) used large amounts of cpDNA and nuclear sequence data to show that *Clermontias.s.* is sister to the purple-fruited clade of *Cyanea*, and that the orange-fruited clade of *Cyanea* is sister to them both.

The corolla of *C.hanaulaensis* is weakly zygomorphic to almost rotate at anthesis with the lobes connate only in the basal half. Although Lammers (1991: 8–9) discussed perianth shape and uses this character throughout his key, these states are often not clear-cut or apparent in herbarium specimens and may depend on the stage at which the flowers were pressed. Photos of mature fresh flowers shows they are at least somewhat zygomorphic in virtually all *Clermontia* species [https://flickr.com/photos/123604592@N07/], even those described by Lammers as being rotate. Field photos of *C.hanaulaensis* flowers show a transition from somewhat zygomorphic to almost rotate as the perianth lobes mature and spread (Fig. 3).

Because gene regions of this new species have not yet been sequenced or included in molecular-phylogenetic studies of *Clermontia* and additional work needs to be done regarding relationships within the genus, our inferences about putative interspecific relationships are based on morphological characters. *Clermontiahanaulaensis* resembles *C.samuelii* C.N. Forbes with two subspecies on east Maui (subsp. hanaensis (H. St. John) Lammers and subsp. samuelii). However, the latter species differs by its smaller leaves with the blade 5–11 cm long and sparsely to densely pubescent beneath, relatively larger flowers with a more strongly curved or arcuate perianth tube 20–38 mm long, shorter less spreading perianth lobes 10–20 mm long, and larger fruits 28–35 mm long and 15–18 mm wide. We have adapted the following key from Lammers (1999) to include *C.hanaulaensis*, which keys out in couplet 10. *Clermontialindseyana* has been considered to occur on Maui based on two poor and hard to identify collections and is included in the key. It is likely that they represent *C.kakeana*, and if so, *C.lindseyana* would be endemic to Hawai‘i Island and does not occur on Maui; nevertheless we have left it in the key.

Several atypical individuals were encountered among the population of *Clermontiahanaulaensis*. One individual has pure white outer and inner perianths that are also shorter and wider than typical for *C.hanaulaensis* (Fig. 3 F, *H. Oppenheimer & K. Severson H82101*BISH, PTBG, US). Two others have purple-pink and white perianth lobes that are shorter and wider than typical for *C.hanaulaensis* (Fig. 3D, E), and in one the calyx tube is slightly separated from the corolla tube (Fig. 3D). These variants were excluded from the circumscription of *C.hanaulaensis* on the basis of their morphology, although we mention them as they may represent hybridization between *C.hanaulaensis* and other sympatric species or between other species in this area. Further study is needed, but is beyond the scope of this paper.

### ﻿Key to the species of *Clermontia* on Maui, adapted from Lammers (1999)

**Table:** 

1	Calyx lobes less than 1/2 as long as corolla, persistent in fruit, distinct or rarely connate at the base, triangular or deltate, rarely oblong or ovate, green	**2**
–	Calyx lobes 1/2 as long to as long as corolla lobes, deciduous in fruit, connate for 1/5–4/5 their length, mimicking corolla in shape, color and texture	**3**
2	Corolla unilabiate, the tube arcuate, 40–60 mm long, the lobes deflexed, 10–20 mm long, 1/5–1/4 the length of the tube	**Clermontiapeleanasubsp.singuliflora**
–	Corolla bilabiate, the tube suberect or curved, 10–36 mm long, the lobes erect or spreading (17–)20–61 mm long, equaling or exceeding tube in length	**4**
3	Corolla, hypanthium, pedicels, peduncle, and petiole muricate; corolla dark rose or occasionally green, the ventral lobes 20–30 mm long	***Clermontiatuberculata* C. Forbes**
–	Corolla, hypanthium, pedicels, peduncle, and petiole smooth; corolla green, the ventral lobes 5–15 mm long	***Clermontiaarborescens*** [two subspecies on Maui]
4	Perianth tubular, the tube curved or arcuate, the lobes 1/5–1/2 as long as tube	**5**
–	Perianth bilabiate or rotate, the tube erect, suberect, or curved, the lobes equaling or exceeding tube in length	**7**
5	Lamina coriaceous, the upper surface glossy; hypanthium hemispheric or obconic, anther tube 13–18 mm long, 3–5 mm in diameter	**ClermontiaoblongifoliaGaudich.subsp.mauiensis (Rock) Lammers**
–	Lamina chartaceous, the upper surface dull; hypanthium turbinate or obovoid; anther tube 10–14 mm long, 2.5–3.5 mm in diameter	**6**
6	Inflorescences pendent, the peduncle (1–) 3–11 mm long; hypanthium 9–19 mm in diameter; perianth 51–85 mm long	***Clermontiagrandiflora*** [three subspecies on Maui]
–	Inflorescences spreading, the peduncle 4–18 mm long; hypanthium 5–10 mm in diameter; perianth 36–55 mm long	***Clermontiasamuelii*** [two subspecies on Maui]
7	Perianth bilabiate	**8**
–	Perianth rotate or at most weakly zygomorphic	**9**
8	Perianth 45–55 mm long, glabrous or sparsely pubescent, the lobes 19–28 mm long; hypanthium 8–14 mm long; anther tube purple or rarely white, 11–14 mm long; lamina chartaceous	***Clermontiakakeana***
–	Perianth 55–65 mm long, pubescent, the lobes 26–38 mm long; hypanthium 12–20 mm long; anther tube white, 17–20 mm long; lamina coriaceous	***Clermontialindseyana***
9	Inflorescence 7–10-flowered	***Clermontiamultiflora***
–	Inflorescence 2–5 (–10)-flowered	**10**
10	Perianth lobes 10–16 mm long, perianth tube 3–5 mm wide	***Clermontiamicrantha***
–	Perianth lobes 20–26 mm long, perianth tube 9–10 mm wide	***Clermontiahanaulaensis***

### ﻿Conservation status

*Clermontiahanaulaensis* should be considered Critically Endangered (CR) due to its limited range and low population numbers (ca. 120–130 individuals), assumed loss and/or decline of most or all of its avian pollinators and dispersal agents, threats such as landslides and treefall, herbivory by alien slugs (*Limaxmaximus*, *Derocrus* spp.) and rats (*Rattus* spp.), and competition with habitat-modifying invasive alien plants including *Ageratinaadenophora* (Spreng.) R.M. King & H. Rob., *Buddleiaasiatica* Loureiro, *Erigeronkarvinskianus* DC, *Melinisminutiflora* P. Beauv., *Psidiumcattleyanum* Sabine, *Rubusrosifolius* Sm., and *Tibouchinaherbacea* (DC) Cogn. *Fraxinusuhdei* (Wenz.) Lingelsh. and *Sphaeropteriscooperi* (Hook. ex F. Muell.) R.M. Tryon are incipient invasive species and are being controlled as encountered. The area was previously impacted by domestic cattle (*Bostaurus*) and feral pigs (*Susscrofa*) which has led in large part to the subsequent alien plant invasion. The progressive upslope spread of axis deer (*Axisaxis*) from areas below the population of *C.hanaulaensis* is of growing concern. Stochastic events such as a hurricane, as well as landslides and treefalls, have the potential to gravely impact this species. Fire is also a threat, although the cumulative effects from climate change are presently unknown.

When evaluated using the World Conservation Union (IUCN) criteria for endangerment (IUCN 2019), *Clermontiahanaulaensis* falls into the Critically Endangered (CR) category, which designates species facing a very high risk of extinction in the wild. The CR designation is indicated when any of the criteria A to E are met. Both Criterion B1 (Extent of Occurrence or EOO) and B2 (Area of Occupancy or AOO) are met with an EOO of less than 100 km^2^ and an AOO of less than 10 km^2^, occurring in a single population of less than the minimum, single 2 km^2^ grid. Criteria A & C address an anticipated decline in population for which there is no data. However, decline is inferred by the lack of robust demographics, with many fewer immature and seedling stages observed compared to mature, reproductive individuals. Criterion D reflects a very limited distribution, currently calculated as a single population of 0.1 km^2^. No quantitative analysis predicting the likelihood of extinction (Criterion E) was conducted. The alphanumeric formula CR B1a, b(v)+B2a,b(iii,v)+C1 represents the current status under IUCN guidelines.

Furthermore, *Clermontiahanaulaensis* should be considered by the US Fish and Wildlife Service as a Candidate for listing as Endangered under the Endangered Species Act of 1973, and a Recovery Plan written, funded, and implemented.

### ﻿Conservation efforts

It is remarkable that this species occurs in a relatively accessible area (no helicopter support is necessary) that has been botanized reasonably well over the past 150 years. Mann & Brigham first collected *Phlegmariurusmannii* (Hillebr.) W.H. Wagner “on the mountains above Ma‘alaea bay” (Hillebrand 1888). Degener collected the type of *Clermontiafurcata* F.E. Wimmer (now a synonym of C.arborescenssubsp.arborescens) “*mauka* of McGregor” in 1952 (Degener 1956). Field work was carried out in this area for at least three decades to varying degrees by Robert Hobdy, Steven Perlman, Kenneth Wood, as well as the first author. Botanical survey work has taken place on adjacent ridges, and further search effort is warranted and planned. This species may have the most restricted range of any taxon in the genus, occupying an area of only 0.1 km^2^.

The Maui Invasive Species Committee (MISC) has been working to control the incipient *Cortaderiajubata* (Lemoine ex Carrière) Stapf infestation in adjacent areas. The region formerly had feral pigs (*Susscrofa*) that the previous landowner, Wailuku Water Co., along with the Mauna Kahālāwai Watershed Partnership (MKWP), formerly West Maui Mountains Watershed Partnership, has successfully controlled through strategic fencing, at least for the time being. Domestic and escaped cattle grazing on adjacent lands have occasionally entered the study area; this threat has also been mitigated, and cattle are no longer on adjacent lands. Axis deer (*Axisaxis*) range is pushing uphill, and the Division of Forestry and Wildlife (DOFAW) of the Hawai‘i Department of Land and Natural Resources (DLNR) and MKWP have begun to renovate an existing pig fence to exclude deer as well as implementing other strategies to limit the size of the herd (Lance DeSilva, DOFAW, pers. comm.).

While this new species exceeds the threshold of 50 wild individuals required to be a target of the Plant Extinction Prevention Program (PEPP), the population will continue to be monitored in case it undergoes a decline. More than 80 mature individual plants have been mapped with GPS, flagged and tagged to assist monitoring and ensure genetic representation *ex situ*. Almost 26,000 seeds from 20 individual plants have already been collected and are in storage at the Lyon Arboretum Seed Storage Laboratory in Honolulu. The PEP Program (http://www.pepphi.org/) strives to collect seeds or cuttings from every individual plant on the USFWS endangered species list, with *ex situ* seed storage, propagation of nursery stock, restoration outplantings into appropriate habitat, and living collections being the main conservation goals.

## Supplementary Material

XML Treatment for Clermontia hanaulaensis

## ﻿Additional information

### Author ORCIDs

Hank Oppenheimer https://orcid.org/0000-0002-9416-1889

David H. Lorence https://orcid.org/0000-0002-6735-9531

Warren L. Wagner https://orcid.org/0000-0001-5012-8422

### Data availability

All of the data that support the findings of this study are available in the main text or Supplementary Information.
